# Substance use and risky sexual behaviours among sexually experienced Ghanaian youth

**DOI:** 10.1186/1471-2458-12-571

**Published:** 2012-07-29

**Authors:** David Doku

**Affiliations:** 1 Department of Population and Health, University of Cape Coast, Cape Coast, Ghana

**Keywords:** Sexual behaviour, Adolescents, Aggregation of risky behaviours, Ghana, Socioeconomic status

## Abstract

**Background:**

The association between risky sexual behaviours and substance uses among Ghanaian youth were investigated.

**Methods:**

An in-school cross-sectional representative survey was conducted among 12-18-year- old youth in Ghana in 2008 (N = 1195, response rate =90%). Logistic regression analyses were employed to investigate the association between substance use (tobacco use, drunkenness, marijuana use and other drug uses) and risky sexual behaviours (sexual debut, condom use and number of sexual partners).

**Results:**

Of all youth, 25% (28% boys and 23% girls) were sexually experienced. The mean age for first sexual intercourse was 14.8 years (14.4 years for boys and 15.1 years for girls). Among the sexually experienced, 31% had multiple sexual partners. Older age (OR = 3.4, 95% CI = 1.7-3.4) and rural residency (OR = 1.5, 95% CI = 1.1-2.1) were independently associated with sexual debut while only older age (OR = 2.4, 95% CI = 1.7-3.4) was associated with condom use. Additionally, smoking (OR = 3.7, 95% CI = 2.0-6.8), tawa use (OR = 2.4, 95% CI = 1.3-4.7), tobacco use (OR = 2.8, 95% CI = 1.7-4.7) drunkenness (OR = 1.7, 95% CI = 1.1-2.8) and marijuana use (OR = 3.3, 95% CI = 1.6-7.0) were independently associated with sexual debut. Furthermore, all substance uses studied were associated with having one or multiple sexual partners.

**Conclusion:**

Substance use seems to be a gateway for risky sexual behaviours among Ghanaian youth. Public health interventions should take into account the likelihood of substance use among sexually experienced youth.

## Background

In most regions of the world (where information is available) the proportion of adolescents who have had sexual intercourse before marriage is high [1]. Risky sexual behaviours, including early sexual intercourse, unprotected sex, multiple sexual partners and non-contraception use can expose adolescents to sexually transmitted diseases (STDs); *e.g.* HIV infection and early pregnancy [2].

Apart from HIV infection, population explosion due to high birth rate in sub-Saharan Africa is a global public health concern. Because adolescents constitute the largest percentage of people in the developing countries, especially in sub-Saharan Africa [3], promotion of safe sex and encouragement of contraceptive use would contribute immensely to the reduction in sex-related morbidity and mortality caused by teenage pregnancy, abortion, HIV/AIDS *etc.* and at the same time reduce the population explosion. To do this however, there is the need to understand their sexual behaviours in order to design effective interventions.

Analogous to what pertains in many sub-Saharan African countries, in Ghana several cultural, economic and social factors affect adolescent reproductive health. These include limited reproductive health services, gender imbalance in sexual decision making, female genital mutilation and male favoured polygamy. In the Ghanaian cultural context, sex before marriage is forbidden. Traditionally, sex education by families is given to only girls, usually by their mothers or an elderly woman in the family during puberty rites. Furthermore, sex is regarded as sacred and sexual issues are hardly discussed in public. Consequently, although sexual health promotion including HIV/AIDS prevention in Ghana promotes abstinence, being faithful to one’s partner and the use of condoms, the former is most emphasised because of religious and cultural values. The extent to which these cultural, religious values and abstinence messages promote delay of sexual debut among young people as well as adolescent sexual behaviours in general is less known.

Health compromising behaviours such as substance use can be regarded as problem behaviour [4,5] because they constitute a deviation from conventional behaviour. Moreover, these behaviours have been reported to co-occur [6,7]. The association between substance use and risky sexual behaviours has often been discussed from two main theoretical perspectives. One school of thought argues that both risky sexual behaviour and substance use are examples of risk-taking behaviours and constitute deviance that share common causes [8-10]. Thus, the relationship between substance use and risky sexual behaviour is spurious [9,10]. Others hold the view that substance use precedes risky sexual behaviour, because, for instance, people tend to have risky sex when under influence of substance use or because they exchange sex for drugs [11]. Consequently, the latter school concludes that substance use acts as a gateway for sexual behaviour. Overall, the relationship between sexual behaviours and other problem behaviours, including substance use among adolescents have been less explored. Studies from developing countries in particular are scarce. The present study investigates the association between substance use (tobacco use, drunkenness, marijuana use and other drug uses) and risky sexual behaviours (sexual debut, condom use and number of sexual partners) among Ghanaian adolescents.

## Methods

A cross-sectional survey was conducted in 2008 on health behaviours and lifestyles of school- aged adolescents in two out of the three administrative zones in Ghana. Thirty schools were randomly sampled, ten per region, from Eastern (total number of schools in the region = 2924), Greater Accra (total number of schools in the region = 1825) and Volta Regions (total number of schools in the region = 2184) in the two zones. The Ghana Education Service's School Health Programme registers of schools in the country was the source of the sampling frame. The sampling was done as follows: First, ten schools were randomly selected so that they comprised of four public Junior High Schools (total number in the three regions = 5325), two private Junior High Schools (total number in the three regions = 1395), three public Senior High Schools (total number in the three regions = 171) and one private Senior High School (total number in the three regions = 47) in each region in order to reflect the school types in Ghana. Second, in each school, all students whose names were found in the class attendance register of the randomly selected classes were eligible to participate in the survey. The eight page questionnaire was anonymous and self-administered and was tested with an initial pilot sample of 50 children in three schools. It was designed to exclude any information that will reveal the identity of the participants. One trained supervisor was assigned to each classroom during the questionnaire administration. The survey commenced simultaneously in all the participating classes in a given school to prevent contamination. Participants were asked to drop their questionnaires in an envelope placed in front of the class on completion. The present study includes all students aged 12-18-year-old (N = 1195, response rate =90% based on school attendance registers). The study protocol was approved by the ethical committee of the Ghana Health Service Research Unit in Accra, Ghana.

### Sexual behaviour and contraception use

The following questions were used to assess the sexual behaviour and contraception use. Have you ever had sexual intercourse? No (go to question xx) Yes. How old were you when you first had sexual intercourse? years old. During your life, with how many different partners have you had sexual intercourse? 1 person, 2 persons, 3 persons, 4 persons or more. This was categorised into three variables i.e. “none” (having no sexual partner), “one” and “multiple” sexual partners. What kind of contraception did you use in your most recent sexual intercourse? Condom, Oral contraceptives, Condom and oral contraceptives, I did not use any contraception, Other, what?

### Indicators of socioeconomic status

Based on previous studies *e.g.*[12,13], various socioeconomic variables were controlled for. Additionally, the demographic variables, age, gender and place of residence were used as background variables. An adolescent’s socioeconomic status (SES) was conceptualised in two dimensions: first, familial SES consisted of factors that described the family (social class of origin) measured by material affluence scale and family structure, and second, factors that predict individual anticipated future social position measured by school performance and plans whether to continue schooling as described below.

### Familial socioeconomic indicators

Three categories of indicators were used to assess the familial socioeconomic status of adolescents as below.

A *material affluence scale* consisting of five categories (poorest, poor, average, affluent and most affluent) was used based on a previous research [14], several indicators were used to construct material affluence scale (MAS). These indicators covered three aspects of material circumstances: household assets and housing characteristics; other assets and school related indicators [15]. Material affluence measures the availability of the resources and goods necessary for decent living in relation to what is generally available in the society [16]. Various kinds of scales measuring material affluence have been constructed to capture the amount of these kinds of resources available in the families [14,17] The items of the scales are not only meant to envelop the key aspects of wealth as well as the material circumstances of the family but are also relatively easy to obtain from adolescents. The relevance of material affluence scale as a proxy measure of affluence is very vital, especially in developing countries where official statistics on socioeconomic indicators may be lacking.

*Family structure* was measured in four categories: nuclear family, both parents alive but not living together, only one parent alive, or both parents dead. Dichotomous variables “nuclear family” and “non-nuclear family” (those living in families other than nuclear family) were made for family structure.

### The adolescent’s individual social position

Adolescents indicated their *school performance* in the previous term examination. These were coded into three categories: above average (excellent, very good), average (good), and below average (average, poor).

Adolescents indicated their *plans after graduation* from the current level of schooling: continue schooling, learn a trade, look for job and not sure. These were coded as continue schooling and not continue schooling (learn a trade, look for job or not sure).

### Indicators of substance use

*Drunkenness* refers to having gotten drunk once a week or more often or about once or twice a month or less often.

*Marijuana users* were those who had ever used marijuana.

*Other drug users* were those who had ever used drugs such as cocaine, LSD, blue-blue (local name for valium 10 mg) or heroin.

*Smoking* was assessed by the question, “Have you ever tried cigarettes or any other tobacco product?” The response options were “No” and “Yes, which?” In this study *smokers* were adolescents who responded “yes” to this question (excluding those who mentioned smokeless tobacco product).

*Tawa* (traditional smokeless tobacco) use was assessed by the question, “Have you ever tried snuff (tawa) and how many times altogether?” The response options were, “No”, “Yes, I have tried once”, “I have used snuff 2–50 times”, “I have used snuff more than 50 times”. In this study, *tawa users* were those who chose any of these responses other than “No”.

Tawa is an indigenous smokeless tobacco that comes in two forms: *Fine-grain tawa*-tobacco that often comes in teabag-like pouches that users "pinch" or "dip" between their lower lip and gum, allow it sit to there and spit out the juice and *chewing tawa***-** tobacco which comes in shredded or twisted tobacco leaves that users put between their cheek and gum, chew it and spit out the juice. *Tobacco users* were those who reported to have smoked or use tawa.

#### Statistical analyses

Pearson’s Chi-square tests (at p < 0.05 statistical significance level) was used to test for the associations between the studied variables- socioeconomic indicators and other health compromising behaviours (tobacco use, drunkenness, marijuana use and other drug use). There were three dependent variables namely ever sexual intercourse (indicating sexual debut), condom use and number of sexual partners. Binary and multinomial logistic regression analyses were used to study the relationship between socio-demographic and substance use indicators, and risky sexual behaviours among adolescents. First, bivariate analyses (Model 1) were computed for each of the explanatory variables, adjusting for age and gender. Second, in a multivariate model, the independent associations between risky sexual behaviours and substance uses were investigated, controlling for various socio-demographic indicators (Model 2). The results were given as odds ratios (OR) and 95% confidence intervals (CI). SPSS package, version 16 SPSS Inc, Chicago, Illinois) was used.

## Results

Of all adolescents, 25% (28% boys and 23% girls) reported having ever had sexual intercourse. About 41.3% of adolescents who were sexually experienced had sexual intercourse before age 15 years. The mean age for first sexual intercourse was 14.8 years (14.4 years for boys and 15.1 years for girls). Among those who were sexually active, 31% have multiple sexual partners (Table 1). The distributions of the SES measures are presented in Table 2. Age at first sexual intercourse was positively associated with the number of sexual partners. Half of those who ever had sexual intercourse used condoms as contraceptive but 31% did not use any contraceptives at all during the most recent sexual intercourse.

**Table 1:** Distribution (%) of sexual behaviour variables by gender

**Sexual behaviour pattern**	**Total (N = 1195)**	**Boys (N = 483)**	**Girls (N = 644)**
**Ever had sexual intercourse (N = 1094)**			
No	74.7	71.6	77.3
Yes	25.3	28.4	22.7
**Age at first sexual intercourse (N = 1164)**			
<11 to 14 years	41.3	44.1	38.7
15 to 17 years	42.6	40.2	45.2
18 years	16.2	15.7	16.1
**Number of sexual partners (1164)**			
1 person	66.3	61.5	70.5
2 persons	14.9	13.8	16.5
3 persons	7.6	10.3	6.2
4 persons or more	10.6	14.4	6.2
**Contraceptive use (N = 1165)**			
Condom	51.5	53.8	49.7
Oral contraceptives	9.4	10.7	8.1
Condom and oral contraceptives	4.4	3.0	5.8
No contraceptives	31.2	30.0	32.4
Other kind	3.5	2.4	4.0
**Smoking (N = 1079)**			
No	93.4	92.0	95.3
Yes	6.6	8.0	4.7
**Tawa use (N = 1106)**			
No	94.3	92.7	96.1
Yes	5.7	7.3	3.9
**Tobacco use (N = 1151)**			
No	90.9	88.5	93.6
Yes	9.1	11.5	6.4
**Drunkenness (N = 1051)**			
Less often or never	87.8	85.5	89.8
Often (once or twice a week or			
often)	12.2	14.5	10.2
**Marijuana use (N = 1163)**			
No	92.8	91.0	94.9
Yes	7.2	9.0	5.1
**Other drug use (N = 1160)**			
No	94.8	93.2	95.4
Yes	5.2	6.8	4.6

**Table 2:** The distribution (%) of the socioeconomic measures of the study subjects by gender

**Variable Material affluence scale (N = 1126)**	**Boys (N = 498)**	**Girls (N = 659)**
Poorest	21.9	16.5
Second poorest	23.1	14.7
Average	20.5	19.3
Affluent	13.9	21.9
Most Affluent	15.7	21.2
Missing	5.0	6.4
**Family structure (N = 1189)**		
Both parents dead	5.4	2.0
Only one parent alive	17.9	16.7
Both parents alive but not living together	21.9	21.2
Nuclear family	54.4	59.5
Missing	0.4	0.6
**School performance (N = 1188)**		
Below average	8.6	16.5
Average	48.4	54.2
Above average	42.2	28.8
Missing	0.8	0.5
**Plans after graduation (N = 1186)**		
Won’t continue schooling	14.3	10.2
Continue schooling	84.3	89.5
missing	1.4	0.3

### Relationship between subtance use and risky sexual behaviours

Smoking, Tawa use, tobacco use, drunkenness, marijuana use and other drug use were all associated with sexual debut and number of sexual partners at the bivariate level (Table 3, Model 1). On the contrary, none of these substance use measures were associated with condom use. In multivariate analyses, after adjusting for the socio-demographic factors, smoking (OR = 3.7, 95% CI = 2.0-6.8), tawa use (OR = 2.4, 95% CI = 1.3-4.7), tobacco use (OR = 2.8, 95% CI = 1.7-4.7) drunkenness (OR = 1.7, 95% CI = 1.1-2.8) and marijuana use (OR = 3.3, 95% CI = 1.6-7.0) were all independently associated with ever sexual intercourse (Table 3, Model 1).

**Table 3:** Odds ratios (OR) and their 95% confidence intervals (CI) of risky sexual behaviours by substance use behaviours among adolescents in bivariate and multivariate analyses

**Health behaviour**	**Ever had sexual intercourse**	**Condom use**	Number of sexual partners*		**Ever had sexual intercourse**	**Condom use**	Number of sexual partners*	
	**Model 1**	**Model 1**	**Model 1**		Model 2**	Model 2**	Model 2**	
	**OR (95% CI)**	**OR (95% CI)**	**OR (95% CI)**	**OR (95% CI)**	**OR (95% CI)**	**OR (95% CI)**	**OR (95% CI)**	**OR (95% CI)**
			**One**	**Multiple**			**One**	**Multiple**
**Smoking**								
No	1.0	1.0	1.0	1.0	1.0		1.0	1.0
Yes	4.1 (2.3-7.2)	1.4 (0.7-2.6)	9.5 (4.6-19.3)	17.7 (8.4-37.3)	3.7 (2.0-6.8)		8.4 (3.7-18.8)	16.5 (7.0-39.0)
**Tawa use**								
No	1.0	1.0	1.0	1.0	1.0		1.0	1.0
Yes	3.7 (2.1-6.7)	1.3 (0.7-2.5)	9.8 (4.7-20.8)	16.6 (7.6-36.3)	2.4 (1.3-4.7)		5.7 (2.5-12.8)	10.4 (4.4-24.6)
**Tabocco use**								
No	1.0	1.0	1.0	1.0	1.0		1.0	1.0
Yes	3.5 (2.2-5.5)	1.3 (0.8-2.3)	8.3 (4.7-14.8)	16.4 (9.0-29.8)	2.8 (1.7-4.7)		6.3 (3.3-11.9)	13.7 (6.9-26.9)
**Drunkenness**								
Less often or never	1.0	1.0	1.0	1.0	1.0		1.0	1.0
Often (once or weak or more often)	2.1 (1.4-3.3)	1.4 (0.8-2.4)	2.1 (1.3-3.3)	4.4 (2.6-7.3)	1.7 (1.1-2.8)		1.7 (1.1-2.8)	3.3 (1.9-5.8)
**Marijuana use**								
No	1.0	1.0	1.0	1.0	1.0		1.0	1.0
Yes	4.8 (2.4-9.7)	1.2 (0.6-2.6)	6.5 (2.6-16.2)	24.4 (10.4-57.2)	3.3 (1.6-7.0)		4.5 (1.7-11.6)	17.1 (6.7-43.3)
**Other drug use**								
No	1.0	1.0	1.0	1.0	1.0		1.0	1.0
Yes	2.2 (1.1-4.6)	1.9 (0.8-4.1)	6.0 (2.4-15.1)	20.5 (8.6-48.7)	1.2 (0.6-2.8)		3.0 (1.1-8.2)	9.1 (3.5-24.0)

*Multinomial logistic regression analysis, reference group is none (no sexual partner).

**Adjusted for age, gender, place of residence, material affluence scale, family structure, school performance and plans after graduation.

Statistical significance level was set at p < 0.05.

Likewise, all the substance use measures were independently associated with the number of sexual partners such that if an adolescent smokes, he/she had 8 and 16 folds the chances of having a sexual partner or multiple sexual partners, respectively, compared to a non-smoker. Also, tawa users were more likely to have a sexual partner (OR = 5.7, 95% CI = 2.5-12.8) or multiple sexual partners (OR = 10.4, 95% CI = 4.4-24.6) compared to those who did not use tawa. Tobacco users had higher likelihood to have a sexual partner (OR = 6.3, 95% CI = 3.3-11.9) or multiple sexual partners (OR = 10.4, 95% CI = 4.4-24.6) compared to non-tobacco users. In like manner, those who were often drunk had nearly 2 folds the likelihood of having a sexual partner and 3 folds the likelihood of having multiple sexual partners compared to their colleagues who never got drunk or did so less often. Marijuana use was associated with having a sexual partner (OR = 4.5, 95% CI = 1.7-11.6) or multiple sexual partners (OR = 17.1, 95% CI = 6.7-43.3). Furthermore, having a sexual partner (OR = 3.0, 95% CI = 1.1-8.2) or multiple sexual partners (OR = 9.1, 95% CI = 3.5-24.0) was more likely among other drug users than non-users.

## Discussion

### Main findings

Sexual debut was relatively high among Ghanaian adolescents. There was also a high number of heterosexual behaviour among those sexually active. Contraceptive use was low and age at first sexual intercourse was positively associated with the number of sexual partners. Older age, male gender and rural residency all increased the likelihood of sexual debut and having one or multiple sexual partners among adolescents. The probability of condom use was higher among older adolescents in comparison with the younger ones. Furthermore, tobacco use, drunkenness and other drug use were all found to increase the likelihood of engaging in sex as well as having one or multiple sexual partners.

### Comparison with previous studies

The prevalence of sexual debut found among Ghanaian adolescents in this study was relatively higher compared to those found among Nigeria adolescents (20%) [17] but lower than those reported among 15-year-old adolescents in eight Eastern and Southern African countries (27.3%) [18]). Although the finding that a quarter of adolescents were sexually experienced can be described as high, still, it is likely to be an under estimation given that premarital sex is frown on by the society and rouses negative reactions from parents, teachers and the Ghanaian society at large, similar to what pertains in other Sub-Saharan African countries [1]. Previous study among 20-24-year-old Ghanaians reported that the age at first sexual debut among men was 19.6 years, the highest among that age group in Sub-Saharan Africa [19]. In the present study the age at sexual debut was lower than those reported in the previous study, and likewise the prevalence of sexual debut before the age of 15 years was higher compared to those found among 15-year-olds in thirty European countries [20] and in eight countries in Eastern and Africa [21]. Despite the age differences between the studies subjects, this study found that the age at sexual debut increases with age suggesting that adolescents are initiating sexual intercourse at earlier age.

Only a third of adolescents who indulged in the most recent sexual intercourse prior to the survey used contraceptives of a kind. This means that most of these adolescents are vulnerable to sexually transmitted infections and also at the risk of teenage pregnancy and teenage parenthood.

Older age, male gender and rural residency were found to increase the likelihood of sexual debut and the number of sexual partners among adolescents. This confirms evidence found elsewhere [12,21]. In many countries, particularly in the developing world, including Ghana, societal sexual expectation for boys and girls varies [22]. In most cases, society is more tolerate towards boys sexual debut even during adolescence and whether married or unmarried. Furthermore, boys and men are expected to be heterosexually active while girls and women are expected to keep their virginity until marriage, and heterosexuality or sex outside marriage is forbidden for girls and women. Consistent with previous studies, this study shows that more adolescent boys reported sexually experience, frequent engagement in sexual intercourse and having heterosexual partners than their female counterparts. With regards to the differences in sexual debut and number of sexual partners between rural and urban adolescents, the higher probability among rural compared to urban residents is likely to be explained by the generally poor education and health services in the rural compared to urban settings.

A striking result in this study is that all the other risky health behaviours studied (tobacco use, drunkenness, marijuana use and other drug uses) were associated with sexual debut and the number of sexual partners. This aggregation of health damaging behaviours is consistent with the theory of problem behaviour [5]. Several studies have also shown the clustering of risky behaviours such as physical inactivity, non-wearing of seat belts and unhealthy diet intake among adolescents [7,23]. Among Zambian adolescents castellation of health compromising behaviours was found among adolescents with history of sexual intercourse [24]. Also in South Korean adolescents similar evidences of the co-occurrence of health compromising behaviour were found [25]. This study seems to confirm substance use as a gateway for indulging in other health damaging behaviours, particularly risky sexual behaviour [11]. In the present study, it is unclear whether adolescents engaged in risky sexual behaviour because they were under the influence of substance use or it was as a result of some “third factor” such as the social environment that facilitated the risky sex or both behaviours. Future studies which would explore these pathways will shed more light on the relationship between substance use and risky sexual behaviours. Studies from African countries which would explore the contextual dynamics would be of immense value. There were no statistically significant associations between substance use and condom use. This could be explained by the small number of condom users in the studied population.

#### Study limitations

This study has a number of limitations. The survey was mainly conducted in school hence the findings might be different from what pertains in the entire adolescent population. Nevertheless, the snap shot of non-students reported provides a good overview of what pertains in non- students (results not shown in this study). The cross-sectional nature of the data limits causal inference while the self-reported nature could result in under reporting of the sexual behaviour, especially given that the Ghanaian society generally detest sexual intercourse among unmarried youth. As the sample of students was drawn from a sample of schools, the clustering of students may slightly change the standard error of the estimates, although unlikely to change neither the overall results nor the conclusion reached in this study. On a whole however, this study fill in an important gap in literature and provides useful piece of information for sexual health promotion among young people.

## Conclusion

Sexual debut was relatively high among Ghanaian youth. On the contrary the use of contraception among the sexually experienced was low. Older age, male gender and rural residency increased youths’ chances of sexual debut and having one or multiple sexual partners. Substance use seems to be a gateway for risky sexual behaviours among Ghanaian youth. These evidences suggest that youth are at risk of HIV infection, other STDs as well as unwanted pregnancy with consequent health and social implications. Sexual health promotion which would emphasise not only abstinence but also the condom and the use of other contraceptives are needed to prevent the youth from becoming victims of unsafe or unplanned sex. Such interventions should take into account the likelihood of tobacco use, drunkenness, marijuana use and the use of other drugs among sexually experienced youth.

## Competing interest

The author declares no competing interests.

## Funding

This study was financially supported by the Competitive Research Funding of the Pirkanmaa Hospital District, Juho Vainion Foundation, and the Finnish Cultural Foundation, Central Fund.

## Pre-publication history

The pre-publication history for this paper can be accessed here:

http://www.biomedcentral.com/1471-2458/12/571/prepub
